# Eu-Doped Pyrochlore Crystal Nano-Powders as Fluorescent Solid for Fingerprint Visualization and for Anti-Counterfeiting Applications

**DOI:** 10.3390/ma15072423

**Published:** 2022-03-25

**Authors:** Layla Brini, Ines Bennour, Alessandra Toncelli, Ramzi Maalej, Mohamed Abdelhedi

**Affiliations:** 1 Laboratory of Inorganic Chemistry, Faculty of Sciences of Sfax, Sfax University, Sfax 3018, Tunisia; layla.brini.etud@fss.usf.tn (L.B.); mohamed.abdelhedi@fss.usf.tn (M.A.); 2 Laboratory of Dielectric and Photonic Materials, Faculty of Sciences of Sfax, Sfax University, Sfax 3018, Tunisia; bennourines1990@gmail.com (I.B.); ramzi.maalej@fss.usf.tn (R.M.); 3 Dipartimento di Fisica, Università di Pisa, Largo B. Pontecorvo 3, I-56127 Pisa, Italy; 4 Istituto Nanoscienze CNR, Piazza San Silvestro 12, I-56127 Pisa, Italy; 5 Istituto Nazionale di Fisica Nucleare-Sezione di Pisa, Largo B. Pontecorvo 3, I-56127 Pisa, Italy

**Keywords:** nano-powder, Europium, luminescence, latent fingerprints

## Abstract

Undoped Y_2_Sn_2_O_7_ and Eu^3+^ doped Y_2_Sn_2_O_7_ samples with doping concentrations 7%, 8%, 9%, and 10% are successfully synthesized by the co-precipitation method. A complete structural, morphological, and spectroscopic characterization is carried out. XRD measurements reveal that samples crystallize in the pure single pyrochlore phase and Eu^3+^ ions occupy sites with D_3d_ symmetry. After mechanical grinding, the average crystallite size is less than 100 nm for all compositions. Optical characterization shows emission from the ^5^D_0_ level towards the lower lying ^7^F_0,1,2,3,4_ levels. The CIE color coordinates of all the pyrochlore phosphors are very close to those of the ideal red light. For the visualization of latent fingerprints, different surfaces are tested, including difficult ones (wood and ceramic), with excellent results. All three levels of fingerprint ridge patterns are visualized: core (Level 1), bifurcation and termination (Level 2), and sweat pores (Level 3). Moreover, our nano-powders are used to prepare a stable fluorescent ink.

## 1. Introduction

Fingerprints are valuable information in forensic science because they provide unique and immutable features for human identification [1]. However, at crime scenes fingerprints are not apparent to the naked eye. For this reason, they are called latent fingerprints (LFPs) and need to be made sufficiently visible with specific techniques [2] like single-metal deposition methods [3], fuming, and powder-dusting techniques [4,5,6]. The details that form the ridge pattern are called loops, arches, and whorls and are organized in different levels. While it is easy to detect the first and the second level ridge details, the third level is quite difficult to be identified, especially on some difficult surfaces like wood and ceramic, and this makes the pattern recognition more difficult and time consuming [7,8,9,10]. Moreover, traditional methods for developing fingerprints have some drawbacks, especially low detection efficiency and high toxicity and very few of them permit the visualization of the third level, i.e., sweat pores.

Among the various methods, power dusting is one of the most used thanks to its extreme simplicity and effectiveness [11]. In this case, the use of fluorescent nanoparticles greatly increases the types of surfaces from which a LFP can be detected and the level of details that can be identified. For this reason, the quest for new materials that can enable the efficient visualization of LFPs is still lively. For example, quantum dots have been proposed as fluorescent powders [12]. They show a good contrast, high selectivity, and sensitivity, but they have relatively high toxicity levels. Organic nanophosphors have some advantages in detecting the LFPs, like long luminescence lifetime, narrow emission, and large Stokes shifts [13]; however, they develop weak prints under ultraviolet light or laser light. Rare earth upconversion fluorescent nanomaterials show bright visible emission under infrared pumping and low toxicity values, but the upconversion efficiency is usually low.

Down-converting rare earth doped nanoparticles overcome this limitation. Among the Lanthanide family Europium (Eu^3+^), Terbium (Tb^3+^), Erbium (Er^3+^), Dysprosium (Dy^3+^), Ytterbium (Yb^3+^), and Praseodymium (Pr^3+^) ions have been identified as the most effective down-converting materials that convert ultraviolet light to visible emission and have been proposed for fingerprint visualization. Moreover, pyrochlore nanoparticles, having the chemical compositions of A_2_B_2_O_7_ [14] doped with lanthanide ions, are one of the most promising powder materials to enhance the development of the LFPs due to their high fluorescence capability, chemical stability, and ease of production in nanometric size [15,16]. These crystal nano-powders are expected to work particularly well on raw surfaces such as household woodwork and multi-colored non-porous items where traditional methods are not so efficient. Among the rare earth ions, Europium in Y_2_Sn_2_O_7_ can generate intense red emission under UV excitation [17,18,19] and has already been proposed as an excellent phosphor for while light LED emission [20], display devices [21], and radioluminescence detection [22]. In this work we analyzed the possibility of using Eu-doped pyrochlores for fingerprint detection as well as for the production of luminescent inks.

## 2. Materials and Methods

### 2.1. Synthesis of Phosphor Samples

Undoped Y_2_Sn_2_O_7_ and Eu^3+^ doped Y_2_Sn_2_O_7_ samples with doping concentrations of 7%, 8%, 9%, and 10% were successfully synthesized by co-precipitation method [17] using Yttrium oxide (99.99%), Europium (III) oxide (99.99%), Tin (II) chloride dihydrate (99.99%), ethylene glycol, and chloridric acid purchased from Merck KGaA (Darmstadt, Germany). Stoichiometric amounts of SnCl_2_.2H_2_O, Eu_2_O_3_, and Y_2_O_3_ were dissolved in 1 M HCl solution and stirred for 2 h. Then, 40 mL of ethylene glycol was added and the solution was slowly heated up to 100 °C. Afterwards, 2 g of urea was added and the solution was stirred 2 h at 150 °C. After being cooled to room temperature, the precipitate was collected by centrifugation at 2500 rpm for 15 min, washed two times with acetone, and dried at room temperature. The samples thus prepared were finally calcined at 900 and 1300 °C in air at a heating rate of 10 °C per minute for 5 h. The undoped Y_2_Sn_2_O_7_ nanoparticles were synthesized by similar method taking only the yttrium and tin precursor.

### 2.2. Ball Milling

As-grown samples were ground using FRITSCH (Idar-Oberstein, Germany) Planetary Ball Milling. Samples were put in a zirconium jar with zirconium balls. Samples were ground at 500 rpm speed for 60 min including 5 min for resting.

### 2.3. Characterization

The phase structure of the samples was analyzed using a Panalytical Pro X’Pert MPD (40 kV, 30 mA) diffractometer (Malvern Panalytical, Malvern, United Kingdom) with a Cu Kα (λ = 1.5406 and 1.5444 Å) radiation at room temperature in the range of 10°–70° with a step size of 2θ = 0.02. The crystallographic phases were identified by comparison with the X-ray patterns of the JCPDS database. The crystallographic parameters were refined using the Rietveld-fit program FullProf [23]. The average crystallite size was calculated from the diffraction line width, based on Scherrer’s equation:

$$D=\frac{K\lambda }{\beta \cos\theta }$$

where D is the crystallite size for the (hkl) plane, λ indicates the wavelength of the incident X-rays, and β is the corrected full width at half maximum in radians. Angle θ is the diffraction angle for the (hkl) plane. The error on calculated XRD crystallite size is 0.4 nm.

The hydrodynamic diameter of the powder was measured with the Dynamic Light Scattering technique (DLS) with a Zetasizer Nano series (Malvern Panalytical, Malvern, United Kingdom). Just before the measurement, the powder was dispersed into deionized water and sonicated to separate aggregates of nanoparticles. The sizing was produced in a glass cuvette with round aperture at room temperature. The average particle size was calculated from the autocorrelation function of the intensity of scattered light from the nanoparticles by Malvern Zetasizer nano software.

The morphology of the samples was observed by high resolution field emission scanning electron microscopy performed with a FEI (Hillsboro, Oregon, USA) Quanta 450 FEG system operating in low vacuum.

Photoluminescence (PL) spectra were measured at room temperature under diode laser pumping at 450 nm as an excitation source. Detection was accomplished using a compact spectrometer (AvaSpec-ULS2048L-SPU2, Avantes, Apeldoorn, The Netherlands) equipped with a 600 line/mm holographic grating and a 10 μm slit working in the 390–900 nm range with a resolution of 0.4 nm. All the spectra were corrected for the spectral response of the system using a halogen lamp as blackbody source.

## 3. Results

### 3.1. Structure of the Prepared Samples

**Figure 1:**
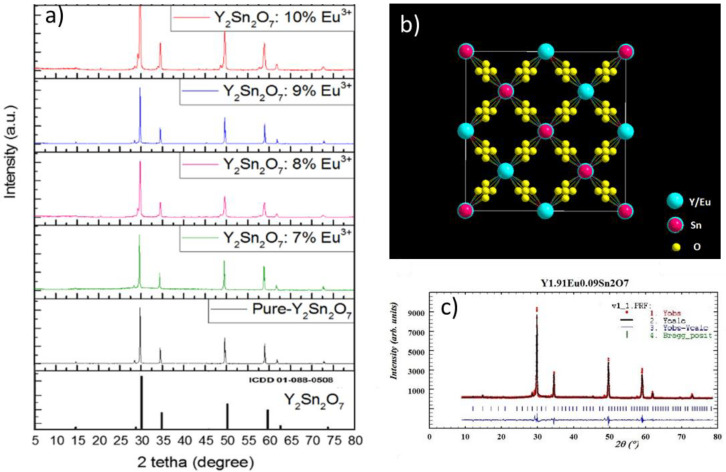
(**a**) Powder XRD patterns of the undoped and doped Y_2_Sn_2_O_7_ samples compared to the JCPDS card for pure pyrochlore structure. (**b**) Crystal structure of doped Y_2_Sn_2_O_7_. (**c**) Fitting results on one of the spectra.

The powder XRD patterns at room temperature of the undoped and doped Y_2_Sn_2_O_7_ samples are depicted in Figure 1a.

Y_2_Sn_2_O_7_ compounds can crystalize in the three different types of crystalline structures, pyrochlore, defect-fluorite, and monoclinic, that can be easily differentiated by XRD analysis [24]. In our case, diffraction peaks are in accordance with the Joint Committee on Powder Diffraction Standards (JCPDS) card No. 01-088-0508, as depicted in Figure 1a. According to these results, Y_2_Sn_2_O_7_ crystallized in pure single pyrochlore phase, and the crystal structure belongs to the cubic crystal system with the Fd-3m space group [21,24,25,26,27]. Moreover, no additional peaks of unreacted SnCl_2_·2H_2_O, Eu_2_O_3_, and Y_2_O_3_ were observed in the XRD-data, indicating that the reaction among the raw materials was complete. The incorporation of Eu^3+^ ions did not modify the XRD-pattern and this, together with the good fitting results, demonstrates that doping with Eu^3+^ does not affect the crystalline structure of the phosphor (Figure 1b). This is not unexpected because the ionic radius of the Eu^3+^ ions (r = 0.95 Å) is similar to that of Y^3+^ ions (r = 0.92 Å); therefore, Eu^3+^ ions can be effectively incorporated into the Y_2_Sn_2_O_7_ host lattice, replacing Y^3+^ ions without distorting the crystal structure.

The cell parameters and the atomic coordinates obtained from a least-square fitting procedure are shown in Table 1. Figure 1c shows an example of the result of the fitting procedure.

The occupancy probability of Y and Eu atoms present in Site A and the Sn atoms present in Site B was refined in such a way that the total occupancy of each site was equal to 1.0000. Five unique crystallographic positions are available in the Wyckoff notation: Y and Eu are disturbed at 16d (1/2, 1/2, 1/2) Wyckoff sites. Sn occupies a single position at 16c (0, 0, 0) Wyckoff sites and there are two positions possible for O at 48f (O) and 8b (O’), while 8a sites are vacant [21]. O’ are in an undisturbed position (3/8, 3/8, 3/8) with respect to the fluorite structure and are tetrahedrally coordinated by Y/Eu cations (Figure 1b). In contrast, O (3/8, 1/8, 1/8) are displaced toward the neighboring vacant 8a sites and are bonded to Y/Eu and Sn. The Y/Eu cations occupy an axially compressed scalenohedron coordinated by six O’ and two O atoms. The cubic crystal structure of the new compound is constructed. The second layer is constructed of one oxygen followed by one Sn atom. The Y and Eu atoms in Site A are coordinated by eight oxygen atoms while the Sn atoms in Site B show a six-fold coordination in which the oxygen atoms occupy the corners of a regular octahedron. The valence values of both cations localized in Site B were calculated from six B-O bond lengths in BO_6_ octahedra, according to Nigham’s work [27]. The two bond-valence with both atoms present in Site B is reported in Table 1.

From the Scherrer equation, we calculated the average crystallite size before mechanical grinding. Results are depicted in Table 2 and compared with the average crystallite size obtained with the DLS technique. The average crystallite size of the samples before grinding was found to be in between 311 and 384 nm with XRD and between 387 and 503 nm with DLS. In all cases, the DLS size is slightly larger than that measured with XRD. This is expected since DLS measures the hydrodynamic diameter that can be slightly larger than the physical dimension of the particle and that can be affected by possible aggregation of the particles.

### 3.2. SEM Characterization

**Figure 2:**
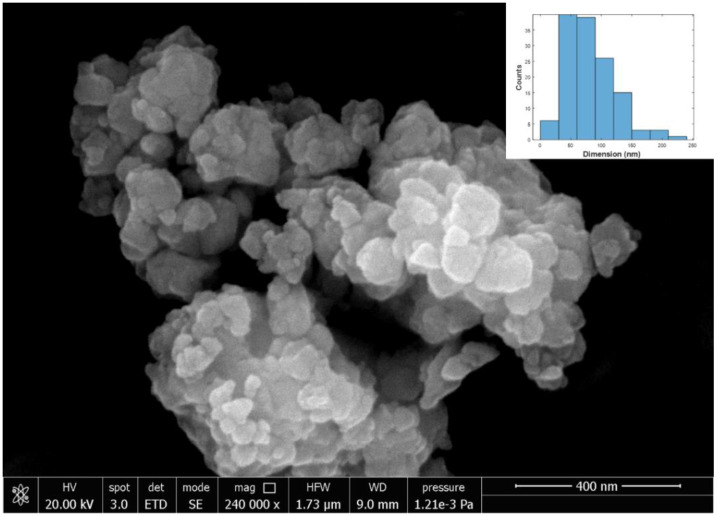
SEM image of 10%Eu: Y_2_Sn_2_O_7_ nanoparticles.

SEM analysis was performed on the samples after grinding. A representative SEM image of the ground Y_2_Sn_2_O_7_:10%Eu sample is shown in Figure 2 together with the corresponding histogram. The formation of very small particles with diameters below 100 nm can be clearly seen. We performed a statistical analysis on the visible particles. Table 3 shows the average mean diameter and standard deviation obtained from the image analysis of the SEM images of the various compositions after grinding. In all cases, the analysis was performed on more than 100 particles and the average diameter obtained was below 100 nm.

### 3.3. Optical Characterization

**Figure 3:**
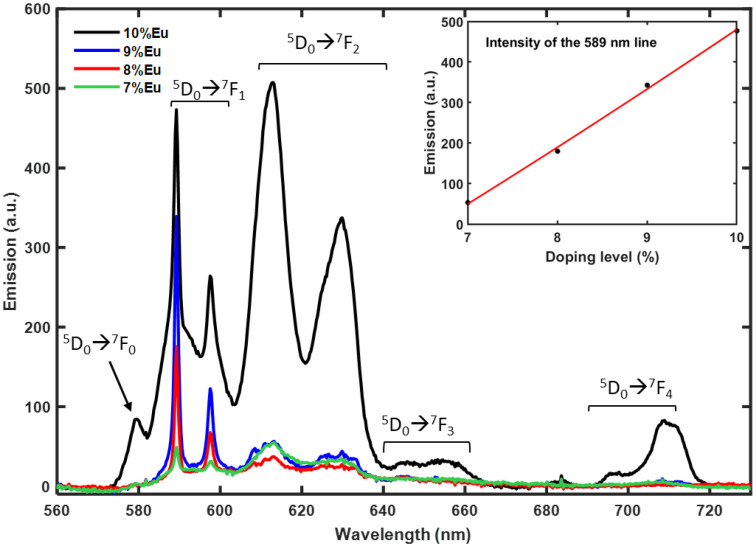
PL intensity of the various compositions under 450 nm excitation.

Figure 3 presents the typical room temperature photoluminescence (PL) spectra of Y_2_Sn_2_O_7_:x%Eu (x = 7, 8, 9, 10) nanophosphors together with the transition assignment. The measured emission spectra are in good agreement with the literature [17,19,21,28,29]. The Eu^3+^ emission comes from several transitions.

All emission channels start from the ^5^D_0_ level towards the lower lying ^7^F_0,1,2,3,4_ levels. Starting from high energy the first transition is the ^5^D_0_ 🡢 ^7^F_0_. Since both levels involved are non-degenerate, it can only give rise to one emission line, but transitions from J = 0 to J = 0 are strictly forbidden in the standard Judd-Ofelt theory. Nevertheless, the occurrence of this transition is a well-known example of the breakdown of the selection rules of the Judd–Ofelt theory due to J-mixing or to mixing of low-lying charge-transfer states into the wavefunctions of the 4f^6^ configuration [30], and it is usually weak in intensity. In our case it is only visible at the highest-doping level, namely 10%Eu with the appearance of a weak peak at 579 nm. The ^5^D_0_ 🡢 ^7^F_1_ can show up to three emission lines because the multiplicity of the ^7^F_1_ level is three, but the D_3d_ point symmetry of the Eu site in pyrochlore structure can only split this level into two components, one of which is doubly degenerate. In fact, we only observed two emission lines at 589.3 nm and 597.5 nm. Moreover, this transition is magnetic dipole in nature, and this means that it is particularly insensible to changes of the environment. For this reason, the intensity of this transition is often considered to be constant and can be used to calibrate the intensity of Eu^3+^ luminescence spectra. We used this line as a probe for the luminescence behavior as a function of the doping level. The result is shown in the inset of Figure 3; the trend is linear and no hints of concentration quenching or other nonlinear energy transfer processes are visible up to the 10% Eu doping level. The ^5^D_0_ 🡢 ^7^F_2_ transition is a so-called “hypersensitive transition”, which means that its intensity is strongly influenced by the local symmetry of the Eu^3+^ ion and the nature of the ligands and this is also the reason why emission lines are broader that in the other transitions. In our case, the 10%Eu doped sample shows a very intense emission from this transition, while it is much weaker at lower doping levels. It is composed by two intense broad bands centered at 612.7 nm and 629.6 nm, one of which is probably a doublet since in D_3d_ symmetry the ^7^F_2_ multiplet is split into three energy levels. The ^5^D_0_ 🡢 ^7^F_3_ can give rise to up to five lines in D_3d_ symmetry, but this is forbidden according to the Judd–Ofelt theory; therefore, it usually produces a very weak emission, as in our case. This band extends from 640 nm to 660 nm and appears as a broad band with some small features. Finally, the ^5^D_0_ 🡢 ^7^F_4_ transition extends from 690 nm to 720 nm and is evident only in the 10%Eu doped sample while it is extremely weak at lower doping levels, like the ^5^D_0_ 🡢 ^7^F_4_ transition. This is not unexpected, because, although not formally considered hypersensitive, this transition is strongly influenced by the symmetry and chemical environment of the Eu^3+^ ions [30].

**Figure 4:**
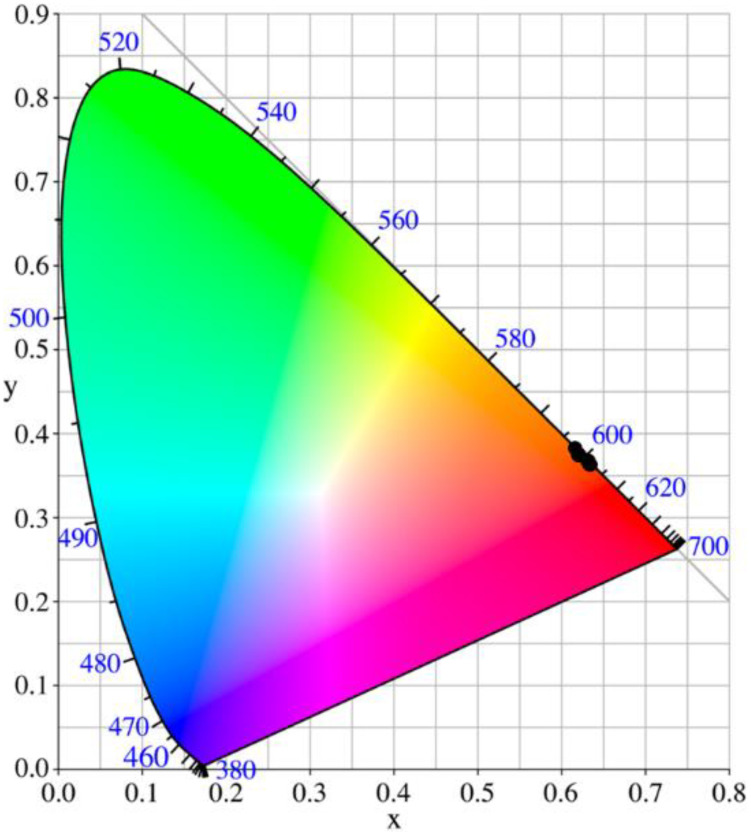
CIE coordinates of the emission of our samples. All samples are located in the red region.

Color coordinates of the phosphors were calculated using The Commission Internationale de l’Eclairage (CIE) 1931 color chromaticity diagram. Figure 4 represents the CIE diagram of Y_2_Sn_2_O_7_:x%Eu (x = 7, 8, 9, 10) nanophosphors. The CIE color coordinates of all the pyrochlore phosphors lie in the range (0.64 ± 0.02, 0.36 ± 0.02), which is very close to that of the ideal red light (0.67, 0.33) [31,32].

### 3.4. Visualization of Latent Fingerprints

**Figure 5:**
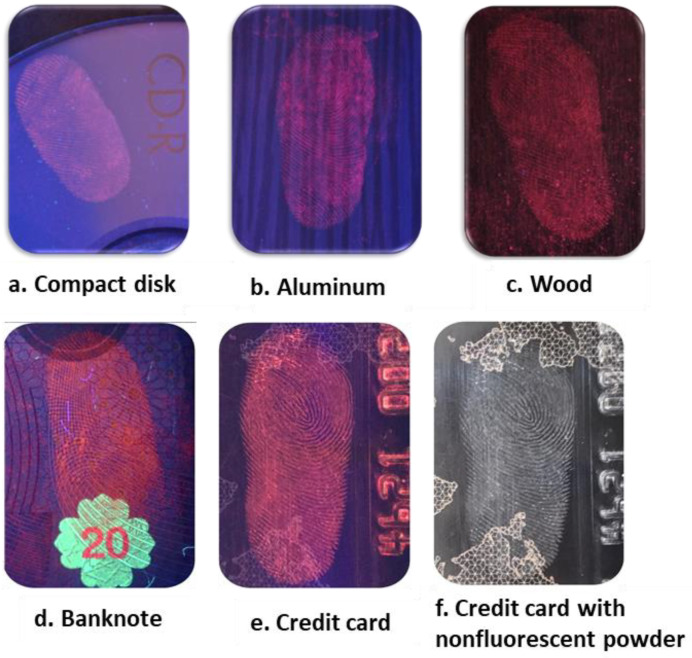

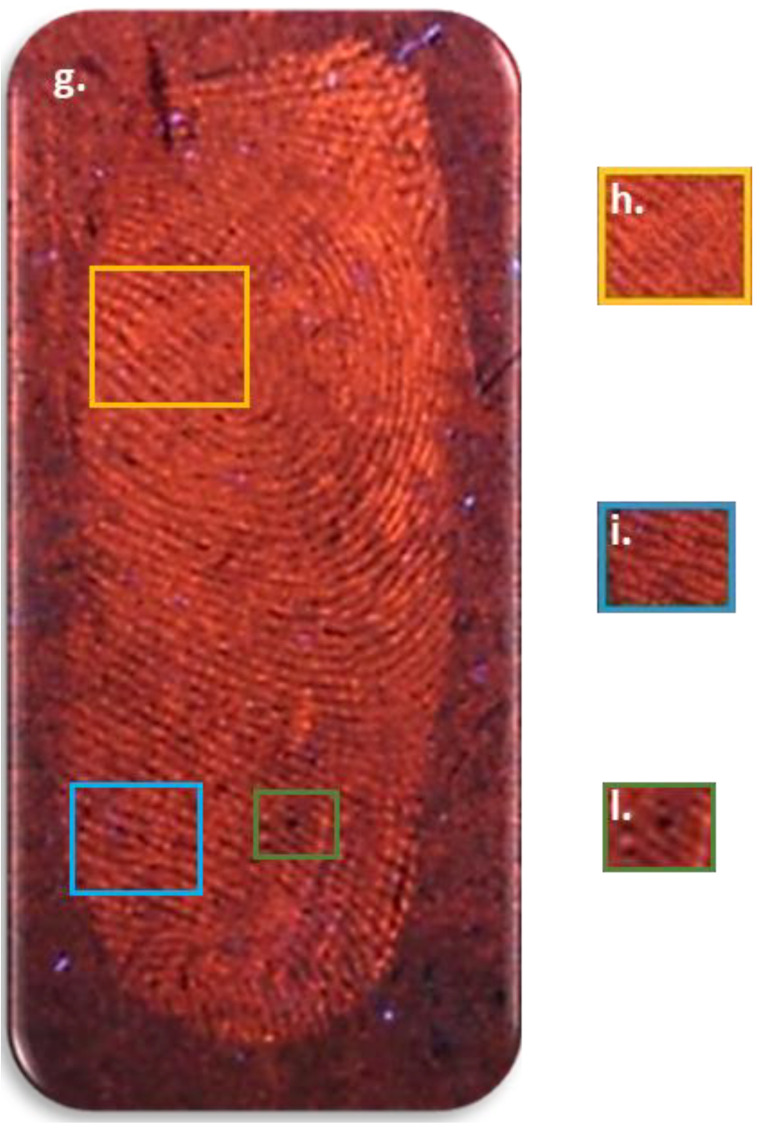
Developed fingerprint images obtained with Eu:Y_2_Sn_2_O_7_ on different surfaces: (**a**) compact disc, (**b**) aluminum foil, (**c**) wood, (**d**) banknote, (**e**) credit card, (**f**) credit card with nonluminescent powder. (**g**) Enlargement of (**c**) with the three levels of fingerprint ridge patterns visualized: (**h**) core (Level 1); (**i**) bifurcation and termination (Level 2); (**l**) sweat pores (Level 3).

In order to test our nanophosphors as LFP development agents, we selected different kinds of surfaces, including porous and non-porous, such as a compact disk, aluminum foil, wood, and ceramic, as shown in Figure 5. After washing and cleaning the donor hands, the fingerprint donor pressed their finger on the different surfaces. Afterwards, the Eu^3+^ doped Y_2_Sn_2_O_7_ nano-powders were stained smoothly over the full area of the LFPs using a feature brush. Then, the excess powder was removed by brushing. Figure 5 shows the fingerprint images developed under a UV lamp at 256 nm, onto a CD, aluminum foil, wood, a banknote, and a credit card. A clear visualization of LFPs was observed on all surfaces; in fact, thanks to the small size of the nanophosphors, the fingerprint ridges are very clear. The finer details of the fingerprints such as the pores were successfully developed, especially on wood and ceramic surfaces, thanks to the shiny red color emitted by these nano-powders. Moreover, Figure 5 also presents a comparison between the fingerprint development on a credit card with luminescent and nonluminescent powder. It can be seen that the nonluminescent powder under ambient light cannot effectively develop the fingerprint pattern on some of the card details, while the whole pattern is clearly visualized under UV illumination.

Figure 5 also shows the enlarged fingerprint pictures developed on wood under UV light. The figure clearly shows that all three levels of fingerprint ridge patterns are visualized: core (Level 1), bifurcation and termination (Level 2), and sweat pores (Level 3). In fact, Level 1 details provide general morphological information such as orientation field, ridge pattern, and fingerprint ridge flow. Level 2 details give information about pattern agreement of the ridges of individual fingerprints. Level 3 details are defined as fingerprint ridge dimensional features, including sweat pores, curvature, and dots [33].

Since the ridges were clearly observed, our new fluorescent samples showed clear patterns with high brightness and contrast to the naked eye on top of the wood, which is considered as a difficult surface. In fact, the red color can be clearly distinguished between brightness of ridges and darkness of furrows.

### 3.5. Application of Luminescent Ink for Detection of Counterfeiting

In recent years, much attention has been devoted to performing fluorescent inks using nanomaterials. However, as far as we know, no serious attempts have been reported to synthesize these transparent and stable Y_2_Sn_2_O_7_: Eu^3+^ colloids for use as fluorescent ink, but the synthesis of stable and transparent luminescent NP colloids in an aqueous medium is a challenging task [34,35,36]. We prepared a luminescent aqueous ink for anti-counterfeiting applications.

**Figure 6:**
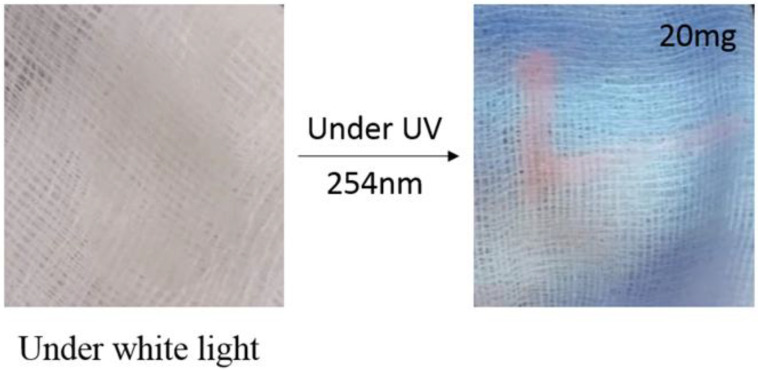
Demonstration of the visibility of our luminescent ink.

In our work, we adapted an encapsulation method in order to produce a transparent and stable Y_2_Sn_2_O_7_: Eu^3+^ colloidal solution from the nano-powders. Initially, 3 g of poly(vinyl alcohol) PVA was dissolved in de-ionized water followed by agitation at 90 °C for half an hour. Then, we added different amounts (10, 20, and 40 mg) of our nano-powders into the previous solution followed by vigorous stirring at room temperature for around 20 min. Finally, we kept the solution under sonification for about 15 min followed by centrifugation to produce highly transparent (>80% in the visible region) luminescent ink. Under ambient conditions, our ink remained stable for about one week without any precipitation. The ink was transparent under visible light but became pink-red in color under UV irradiation. Figure 6 shows the letter L written with the florescent ink on tissue. Under ambient light the ink is invisible, but it becomes visible under UV irradiation, with increasing visibility at increasing amount of nano-powder dispersed.

## 4. Conclusions

In this work, Eu:Y_2_Sn_2_O_7_ nanophosphors were successfully synthesized via the co-precipitation method followed by a further grinding treatment. XRD confirmed the cubic single phase pyrochlore structure of the phosphors. After mechanical grinding, all the samples showed an average dimension of less than 100 nm. Bright red light is emitted by the nanophosphors after UV illumination. This led to an efficient visualization of LFPs on different surfaces like a compact disk, aluminum foil, wood, and ceramic. Moreover, we prepared a stable luminescent ink for anti-counterfeiting applications. We believe these findings can be useful both in forensic science and in anti-counterfeiting applications.

## Figures and Tables

**Table 1:** Atomic coordinates and site occupancy fraction for Y_2_Sn_2_O_7_:9%Eu.

Atom	Site	Symmetry	X	Y	Z
Y/Eu	16d	D_3d_	0.5	0.5	0.5
Sn	16c	D_3d_	0	0	0
O	48f	C_2v_	0.337	0.125	0.125
O’	8b	T_d_	0.375	0.375	0.375

**Table 2:** Average crystallite size.

Composition	Crystallite Size from XRD (nm)	Crystallite Size from DLS (nm)
Y_2_Sn_2_O_7_:7%Eu	384.5	405
Y_2_Sn_2_O_7_:8%Eu	311.6	487
Y_2_Sn_2_O_7_:9%Eu	360.5	503
Y_2_Sn_2_O_7_:10%Eu	326.5	387

**Table 3:** Measured length of the samples.

**Sample**	**Size (nm)**	**Standard Deviation (nm)**
Y_2_Sn_2_O_7_	56	31
Y_2_Sn_2_O_7_:7%Eu	99	57
Y_2_Sn_2_O_7_:8%Eu	57	34
Y_2_Sn_2_O_7_:9%Eu	70	50
Y_2_Sn_2_O_7_:10%Eu	82	39

## Data Availability

The data presented in this study are available on request from the corresponding author.
